# Towards Chagas disease elimination: Neonatal screening for congenital transmission in rural communities

**DOI:** 10.1371/journal.pntd.0005783

**Published:** 2017-09-11

**Authors:** Pamela Marie Pennington, José Guillermo Juárez, Margarita Rivera Arrivillaga, Sandra María De Urioste-Stone, Katherine Doktor, Joe P. Bryan, Clara Yaseli Escobar, Celia Cordón-Rosales

**Affiliations:** 1 Center for Health Studies, Universidad del Valle de Guatemala, Guatemala City, Guatemala; 2 Center for Biotechnology Studies, Universidad del Valle de Guatemala, Guatemala City, Guatemala; 3 Department of Entomology, Texas A&M University, College Station, Texas, United States of America; 4 School of Forest Resources, University of Maine, Orono, Maine, United States of America; 5 University of Miami, Jackson Memorial Hospital, Miami, Florida, United States of America; 6 Centers for Disease Control and Prevention Central America Regional Office, Guatemala City, Guatemala; 7 Division of Global Health Protection, Center for Global Health, Centers for Disease Control and Prevention, Atlanta, GA, United States of America; 8 Distrito de Salud de Comapa, Jutiapa, Ministerio de Salud PuÂblica y Asistencia Social de Guatemala; Institut de Recherche pour le Développement, BENIN

## Abstract

Chagas disease is a neglected tropical disease that continues to affect populations living in extreme poverty in Latin America. After successful vector control programs, congenital transmission remains as a challenge to disease elimination. We used the PRECEDE-PROCEED planning model to develop strategies for neonatal screening of congenital Chagas disease in rural communities of Guatemala. These communities have persistent high triatomine infestations and low access to healthcare. We used mixed methods with multiple stakeholders to identify and address maternal-infant health behaviors through semi-structured interviews, participatory group meetings, archival reviews and a cross-sectional survey in high risk communities. From December 2015 to April 2016, we jointly developed a strategy to illustratively advertise newborn screening at the Health Center. The strategy included socioculturally appropriate promotional and educational material, in collaboration with midwives, nurses and nongovernmental organizations. By March 2016, eight of 228 (3.9%) pregnant women had been diagnosed with *T*. *cruzi* at the Health Center. Up to this date, no neonatal screening had been performed. By August 2016, seven of eight newborns born to Chagas seropositive women had been parasitologically screened at the Health Center, according to international standards. Thus, we implemented a successful community-based neonatal screening strategy to promote congenital Chagas disease healthcare in a rural setting. The success of the health promotion strategies developed will depend on local access to maternal-infant services, integration with detection of other congenital diseases and reliance on community participation in problem and solution definition.

## Author summary

Chagas disease is caused by a parasite transmitted by insects that infest households living in extreme poverty conditions. The parasite can also be transmitted from mother to child during pregnancy. If detected at birth, the infection can be treated effectively with available drugs. However, access to professional neonatal healthcare is limited in rural communities such as those affected by Chagas disease. We developed a strategy to promote access to a simple neonatal diagnostic test in a rural region of Guatemala considered at risk for Chagas disease. The strategy included collaboration between Health Center personnel, midwives and non-governmental organizations that play a local role in maternal-infant care. During the implementation of a health promotion campaign, screening revealed previous infection in almost one of every 25 pregnant women. Most babies born to positive women were tested at the Health Center for parasites in blood. The implementation of similar strategies to prevent congenital Chagas in other rural areas should consider local maternal-infant care practices. This strategy of collaboration between Ministry of Health, community health workers, non-government organizations, academia and external governmental support could be expanded to screen for other diseases, such as Zika, that require early detection to improve overall infant health.

## Introduction

Chagas disease is a vector-borne illness that can also be transmitted congenitally, via blood transfusion, organ donation, lab accidents or ingestion [1]. With the implementation of vector control programs, insect transmission of *Trypanosoma cruzi* has become less common [2] and vertical transmission has increased in importance [3–6]. In Argentina, a prospective study showed that 67.3% of 107 patients enrolled were infected congenitally, while only 4.7% via vector transmission [7]. In 2005, the Guatemalan Ministry of Health (MoH) proposed to include congenital Chagas disease screening and treatment of children. [8]. However, program implementation has been limited by lack of evidence on congenital incidence rates. We are implementing a strategy to screen congenital transmission in populations at highest risk.

Congenital Chagas disease is an acute infection [9] with 27–57% asymptomatic cases in children [10, 11]. The consequences of infection in utero can be seen prior to birth, with spontaneous abortion and stillbirth and, upon birth, neonates have a higher mortality within the first two days [10]. Diagnosis of congenital Chagas can be achieved with varying degrees of sensitivity by screening the newborn´s blood within the first month after birth by microscopy, hemoculture or by polymerase chain reaction [5, 12, 13]. Infants may be screened serologically 10 months after birth, when maternal transplacental antibodies have waned [14]. Some potential risk factors for vertical transmission of Chagas disease include the degree of parasitemia [15–17], the presence of acute infection in the mother [18], and co-infection with HIV [17, 18]. Treatment should be implemented immediately after diagnosis to improve prognosis [19]. The oral treatment must be monitored by trained health personnel due to potential adverse effects [5, 15]. Thus, screening and treatment programs require access to maternal-infant care within an institutional platform.

Over the past five years, we have worked at the municipality of Comapa in the Department of Jutiapa. This is a region of eastern Guatemala that, prior to the launching of the vector control program in 2000, had some of the highest triatomine infestations (>40% infested households) [20] and seroprevalence in school-age children (13.75%) [10] in the country. We extended our previous multidisciplinary study of Chagas disease vector control [21], working in collaboration with the health personnel, communities and non-governmental organizations to establish a congenital Chagas disease healthcare program. This study aimed to improve congenital Chagas disease detection and treatment in this rural area of Guatemala through a multi-stakeholder driven strategy, based on the PRECEDE (Predisposing, Reinforcing, and Enabling Causes in Educational Diagnosis and Evaluation) PROCEED (Policy, Regulatory and Organizational Constructs in Educational and Environmental Development) model [22] for community interventions. After program implementation, newborns are being screened for Chagas disease at the Health Center (HC).

## Methods

### Ethics statement

The study obtained ethical approval from both the Universidad del Valle de Guatemala (#108-10-2014, #100-04-2014) and the Ministry of Health of Guatemala (01–2014) Institutional Review Boards. Individual written consents were obtained from participants before interviews and health access surveys.

### Study site

**Fig 1:**
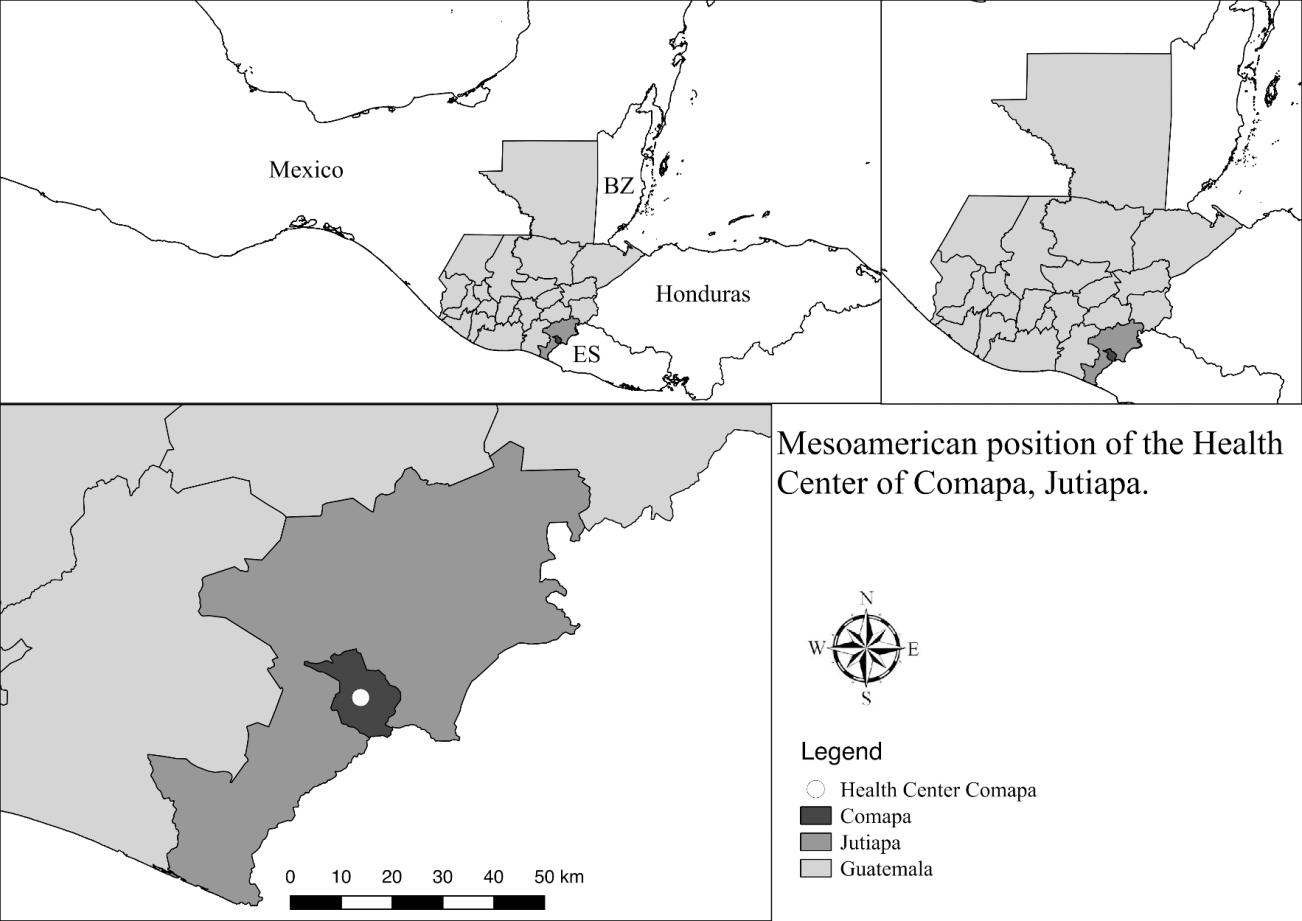
Map of study site location. Comapa municipality and health center location in the department of Jutiapa, Guatemala, relative to Mexico, Honduras, Belize (BZ) and El Salvador (ES). The map was developed using QGis 2.14 with publicly available administrative boundaries.

Comapa is a municipality located in the department of Jutiapa, in the southeastern region of Guatemala bordering El Salvador at -89°54′46.8″ and 14°6′38.6748″ (Fig 1). Comapa was selected to develop the congenital Chagas disease surveillance protocol due to the presence of a newly built maternity ward (2012), the relevance of the disease to local health authorities, an ongoing Chagas diagnosis and treatment program for children and adults, and an incipient Chagas disease prenatal screening program. Prenatal screening includes a rapid diagnostic test (if available) at the HC in Comapa, with the rapid test provided by the non-governmental organization (NGO), Médicos con Iberoamérica (IBERMED), followed by a single ELISA test performed at the Area Laboratory in Jutiapa.

### Applying the PRECEDE PROCEED model

Quantitative and qualitative research methods were used to understand the local socio-ecological system driving health behaviors. Ultimately, we aimed to develop and implement a sustainable community process for the surveillance of congenital Chagas disease. For this, we conducted the situational assessments of all phases of the PRECEDE component of the model: phase 1 (social), phase 2 (epidemiological), phase 3 (educational and ecological) and phase 4 (administrative and policy). We also conducted one phase of the PROCEED component of the model, phase 5 (design and implementation) of the health promotion strategy [22]. We partnered with the MoH and identified stakeholders (midwives, NGOs, Municipal offices, maternal HC and laboratory personnel) throughout the study to ensure the joint identification of the problems and solutions. Table 1 shows the project phases, timeline, activities and stakeholders in chronological order.

**Table 1:** Chronological project timeline and activities according to PRECEDE-PROCEED phases.

Month/Year	Phases	Activity	Stakeholder(s)
January–June 2011	(1) Social assessment	Entomological and KAP´s surveys	Community [21]
September 2012-May 2013	(1) Social assessment	Participatory action research (PAR) meetings	Community [23]
October 2013	(1) Social assessment	Semi-structured interviews and participant observation	Women [24]
March 2014	(4) Administrative and policy assessment	Meetings with Jutiapa Health Area and NGO	MoH and NGO
October 2014	(2) Epidemiological assessment	Semi-structured interviews and participant observation	MoH and midwives
December 2014	(3) Educational assessment	Participatory meetings workshops	MoH and midwives
December 2014	(3) Educational assessment	Participant observation and presentation of results	MoH and midwives
December 2014-January 2015	(3) Educational assessment	Participatory meeting workshops to validate and distribute	MoH and midwives
April-August 2015	(3) Ecological assessment	Maternal-infant health access survey	Women 15–44 years of age
June 2015	(4) Administrative and policy assessment	Archival review of registered deliveries	MoH and pregnant women
April 2016	(4) Administrative and policy assessment	Archival review of laboratory diagnostics	MoH and pregnant women
December 2015-April 2016	(5) Intervention design and implementation	Educational material design, validation and stakeholder training	HC, Vector Control, Women´s Municipal Office and World Vision personnel
April 2016	(5) Intervention design and implementation	Educational material distribution	HC, Vector Control and World Vision personnel
August 2016	(5) Intervention design and implementation	Semi-structured interviews, archival review of deliveries and laboratory diagnostics	HC, Vector Control and World Vision community collaborator

#### Phase 1: Social assessment

In 2011 we conducted entomological and Knowledge, Aptitude, and Practices (KAP´s) surveys in 30 communities of Comapa [21]. These surveys were used to identify Chagas disease risk factors which we used to conduct educational Participatory Action Research (PAR) meetings in nine communities [23]. During these meetings, a partnership was established with a predominantly female population. Thus, eighteen females were selected across six communities for semi-structured interviews to gain in-depth understanding regarding the implementation of daily activities in the vector control project [24].

#### Phase 2: Epidemiological assessment

In October 2014, we conducted semi-structured interviews with 26 midwives from 19 communities and nine HC personnel; including six nurses, the night shift maternity ward doctor, head laboratory technician and a health promoter. The interviews had open-ended questions to identify a workflow with key features of the local Maternal and Child Health practices. Questions included type of attention provided to women, frequency of visits and general profile of pregnant women visiting the facilities. We also registered and analyzed profiles of HC registered midwives (age, community, attention frequency and community care coverage).

#### Phase 3: Ecological and educational assessment

We conducted participatory group meetings between December 2014 and January 2015, in collaboration with ten midwives and three HC personnel. The HC personnel that collaborated were those involved in maternal and child healthcare practices in the region. Midwives that collaborated were those that had assisted ≥ 6 home deliveries/month, worked in more than one community and actively participated during the previous three midwife training workshops led by the HC. The information used to select our collaborators was obtained from semi-structured interviews and HC records. The meetings consisted of workshops addressed to either the HC personnel or the midwives. During these workshops we jointly developed culturally appropriate promotional material for congenital Chagas disease screening. The workshop for the midwives consisted of five stations, each with a specific theme: parasite microscopic morphology, how to recognize a person with *T*. *cruzi* infection, midwife self-representation, healthy mother, and healthy baby perception. Midwives were asked to work in pairs rotating from one station to another in which they had to draw the concepts previously presented. Posters to promote neonatal screening were generated based on the self-representation of the midwives and nurses, and validated with participant feedback.

We also conducted a maternal-infant health access survey between April and August 2015, among women 15–44 years of age. We selected the same households from our 2012 study where a female was available for interview [23]. Households where women between 15 and 44 years of age were not available were consistently replaced with the house to the right (n = 490). The health access survey was a face-to-face structured questionnaire consisting of closed-ended and semi closed-ended questions. The questions were related to knowledge regarding Chagas disease, triatomines, health services, and maternal-infant health care practices. The questionnaire was field validated prior to implementation. The recorded information was entered to an SPSS 20V (IBM, USA) database for descriptive analysis. Data are available in S1 Appendix.

#### Phase 4: Administrative and policy assessment and intervention alignment

Based on stakeholder analysis from our previous study [23], we conducted several meetings in March 2014 with different branches of the MoH (Hospital head, epidemiologist, laboratory diagnostics, vector control unit) and IBERMED to define the desired strategy for Chagas disease surveillance and management in Comapa.

Between April and October 2014 we conducted interviews with Comapa´s HC maternity ward nurse and laboratory technician. We determined the ongoing activities for prenatal and congenital Chagas disease screening; and the technical capabilities and diagnostic practices in Comapa. In June 2015, we reviewed archival records for deliveries registered at the HC, in collaboration with the head nurse. In April 2016, we reviewed the prenatal Chagas screening performed from November 2015 to March 2016, in collaboration with the HC technician.

#### Phase 5: Intervention design and implementation

Based on the situational analysis of the PRECEDE, and responding to the key factors influencing the health behavior, we designed and implemented a stakeholder-driven intervention that included: (1) establishment of a collaborative partnership between government, non-governmental organizations, university, and community groups; (2) promotional material for midwives to refer pregnant women and their newborns to the HC for diagnostics, (3) a community-based communication campaign; and (4) a protocol to train stakeholders on the proper use of the educational material. The communications campaign included the planning, pretesting, implementation, and assessment of the strategy. We developed educational material (flipchart and banner) describing vectorial and congenital Chagas disease transmission, detection, treatment and prevention, based on an Argentinian promotional campaign [25] and images from “Casa Limpia Patio Limpio” developed by the MoH and the Japanese International Cooperation Agency [26]. We pre-tested the flipchart and banner for attractiveness, comprehension, acceptance and engagement with local communities. We trained personnel from World Vision, the Women´s Municipal Office and MoH on the use of the flipchart. We conducted a preliminary evaluation of the implementation through participant observation during three community meetings.

## Results

### PRECEDE

#### Phase 1: Social assessment to develop a congenital Chagas disease screening program

The socioeconomic factors obtained from the entomological and KAP´s surveys from 2011 showed that households that received information from the HC were at a lower risk of having triatomine infestation [21]. This prompted the hypothesis that the population with higher risk of infection would have lower access to congenital Chagas disease screening. In addition, study participants from the PAR meetings belonging to communities with persistent infestation recommended to include information about the disease, transmission routes, diagnosis and treatments as critical components for future Chagas disease control strategies [23]. Finally, in-depth female interviews showed that women have a cultural role in childcare and family health issues [24].

#### Phase 2: Epidemiological assessment to identify the extent of the health problem

Interviews confirmed that Chagas disease treatment was available at the Health Center, making decentralized neonatal treatment feasible. A close relationship between the HC and midwives, revealed that the MoH has a policy requiring all practicing midwives to attend periodic training at their local HC, as a pre-requisite to register birth certificates. In 2014, there were 40 registered midwives that received monthly training and had their deliveries registered at the Comapa HC. Thus, it was deemed critical to identify and work with midwives as key stakeholders to develop a congenital disease-screening program. Interviews with the nurses and laboratory technician indicated that obtaining heel prick blood samples and subsequent parasitological screening through microscopic analysis of a microhematocrit capillary, were feasible and could be implemented for neonatal screening.

#### Phase 3: Educational assessment to identify enabling and reinforcing factors that affect maternal infant health practices

During the participatory group meetings, we relied on the use of images instead of text so the HC personnel and especially the midwives, had a more efficient understanding and greater retention of information. The meetings included information regarding previously identified risk factors for *T*. *dimidiata* infestation [21], the importance of midwives and HC personnel for effective screening for *T*. *cruzi* infection, and audio-visual workshops to exchange knowledge of the disease. The audio-visual workshops were used to design and develop posters to promote community-based Chagas disease neonatal screening. Most of the midwives had difficulty drawing; as a consequence, they had to interpret their work after each activity. We sought to include all factors and traits for the production of promotional material; such as the way midwives visualize themselves, the nurses and a healthy baby.

**Fig 2:**
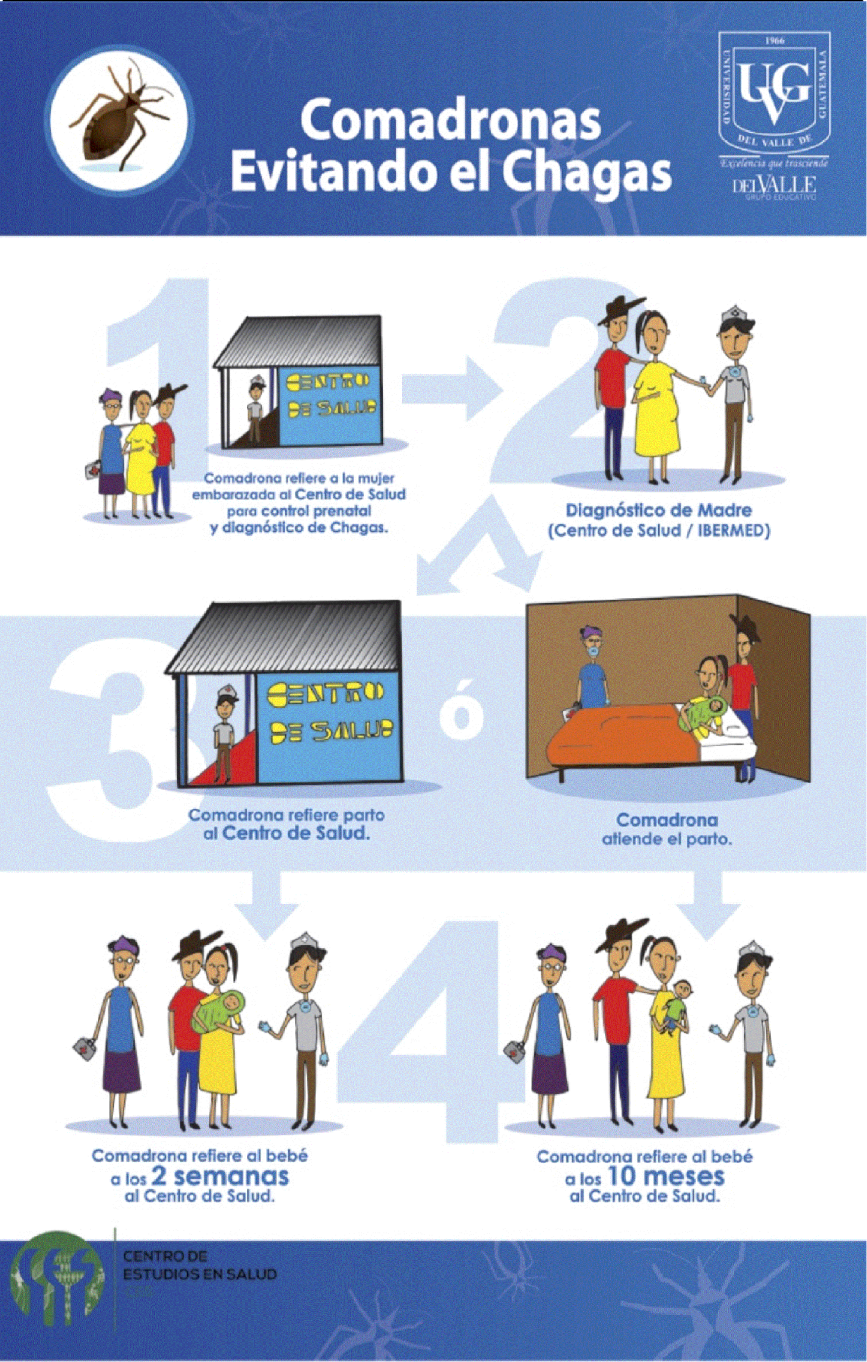
Promotional poster “Midwives preventing Chagas”. The poster was developed through participatory activities with midwives to show the steps to promote congenital Chagas disease screening.

The resulting poster to promote screening for early detection of Chagas Disease (Fig 2) is titled “Midwives preventing Chagas” (“Comadronas Evitando el Chagas”, in Spanish). The first step shows (1) the midwife referring the pregnant woman to the HC nurse for screening. Step two shows (2) the HC nurse performing diagnosis in collaboration with rapid tests donated by IBERMED. Step three shows (3) the midwife referring the pregnant woman to the HC for delivery or, after delivering at home, referring the baby for diagnosis at the HC (4) during their first vaccination at two weeks (2 semanas) or the measles mumps rubella vaccination at 10–12 months (10 meses).

**Fig 3:**
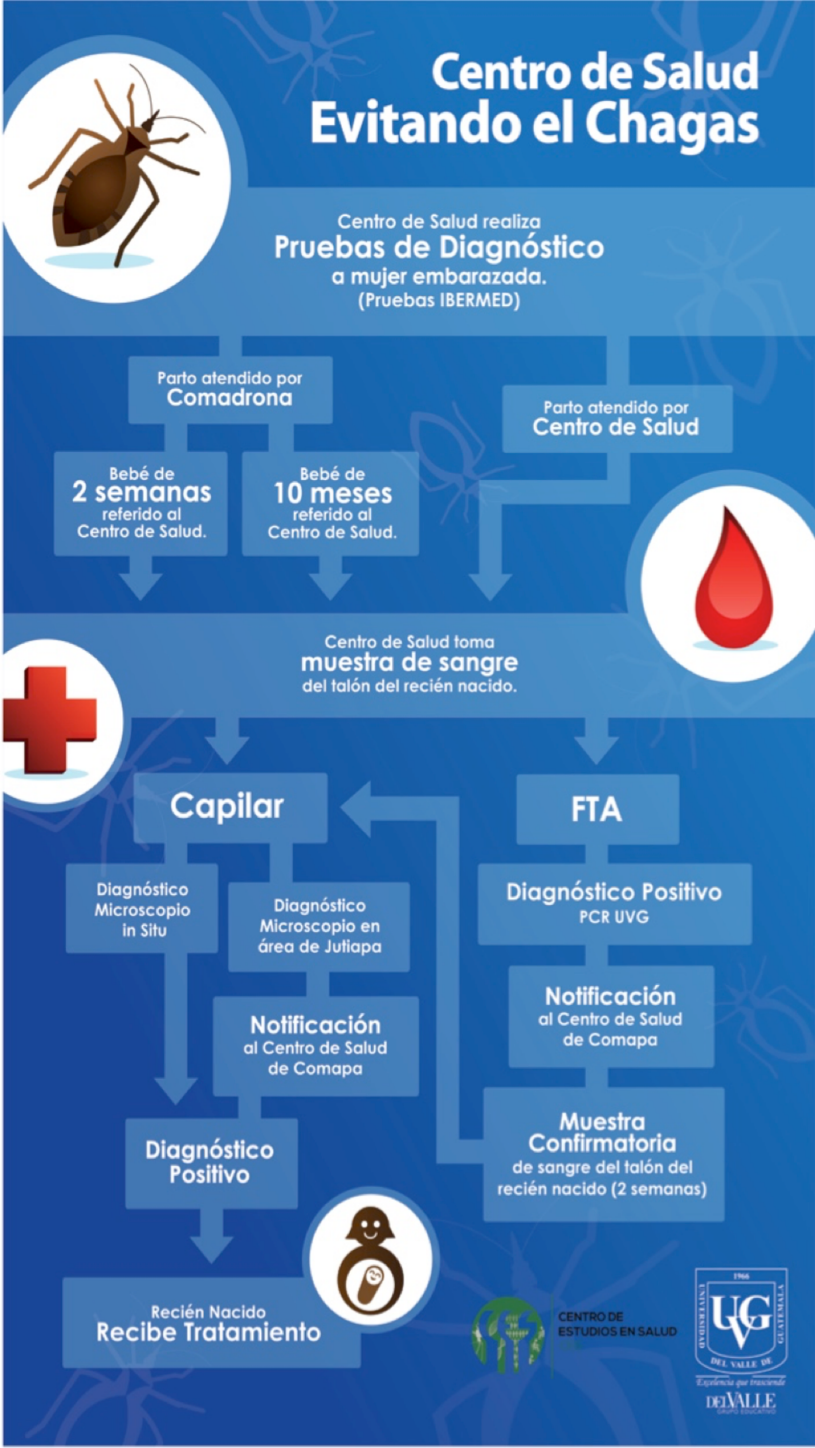
Promotional poster titled “Health Center Preventing Chagas”. The workflow and poster were developed through participatory activities with Health Center personnel to promote screening for early detection of congenital Chagas disease at the HC. The workflow shows two parallel arrows for the same newborn heel prick blood sample. The blood sample for buffy coat separation in capillary tube is taken simultaneously with the blood sample preserved in nucleic acid preservation cards (FTA) for PCR analysis. The arrow connecting the PCR result with the capillary result indicates confirmation of the microscopy, to optimize the parasitological screening method at the Health Center. Children born to positive mothers, but that are not screened within two weeks of birth, should be brought to the Health Center at ten months for antibody rapid test only.

The HC personnel helped develop the technical workflow (Fig 3) for congenital screening in the maternity ward. Titled “Health Center Preventing Chagas” (Centro de Salud Evitando el Chagas, in Spanish), it shows the steps required for congenital disease screening, starting with the diagnostic of pregnant women at the HC with rapid tests provided by IBERMED and confirmed by ELISA. Subsequently, two options and two ways to proceed: if a delivery is assisted by the HC or by a midwife, the newborn should be taken to the HC before two weeks (preferred for parasite test), or after ten months for antibody test only. A parasitological diagnostic test is recommended for newborns born to a positive woman through parasitology of heel prick blood sample collected in a capillary [5, 9, 12, 13]. Molecular diagnosis is performed to confirm the parasitological result only during method optimization at the Health Center [27]. Polymerase chain reaction (PCR) of heel prick blood collected on nucleic acid collection cards (FTA), to be performed at Universidad del Valle de Guatemala (PCR UVG). If the child was born at the HC, the same procedure is to be performed while mother and child are still at the HC. The final result is to prompt treatment of parasitologically positive neonates.

In December 2015, we distributed promotional material to all midwives and the HC personnel. The printed posters were presented to midwives and HC personnel during a meeting where each participant (midwives and HC personnel) had to interpret and explain to the group the steps represented in the posters. Subsequently, participants presented the material to a new group of midwives at one of the monthly training sessions at the HC.

#### Phase 3: Ecological assessment to identify enabling and reinforcing factors that affect maternal infant health practices

**Fig 4:**
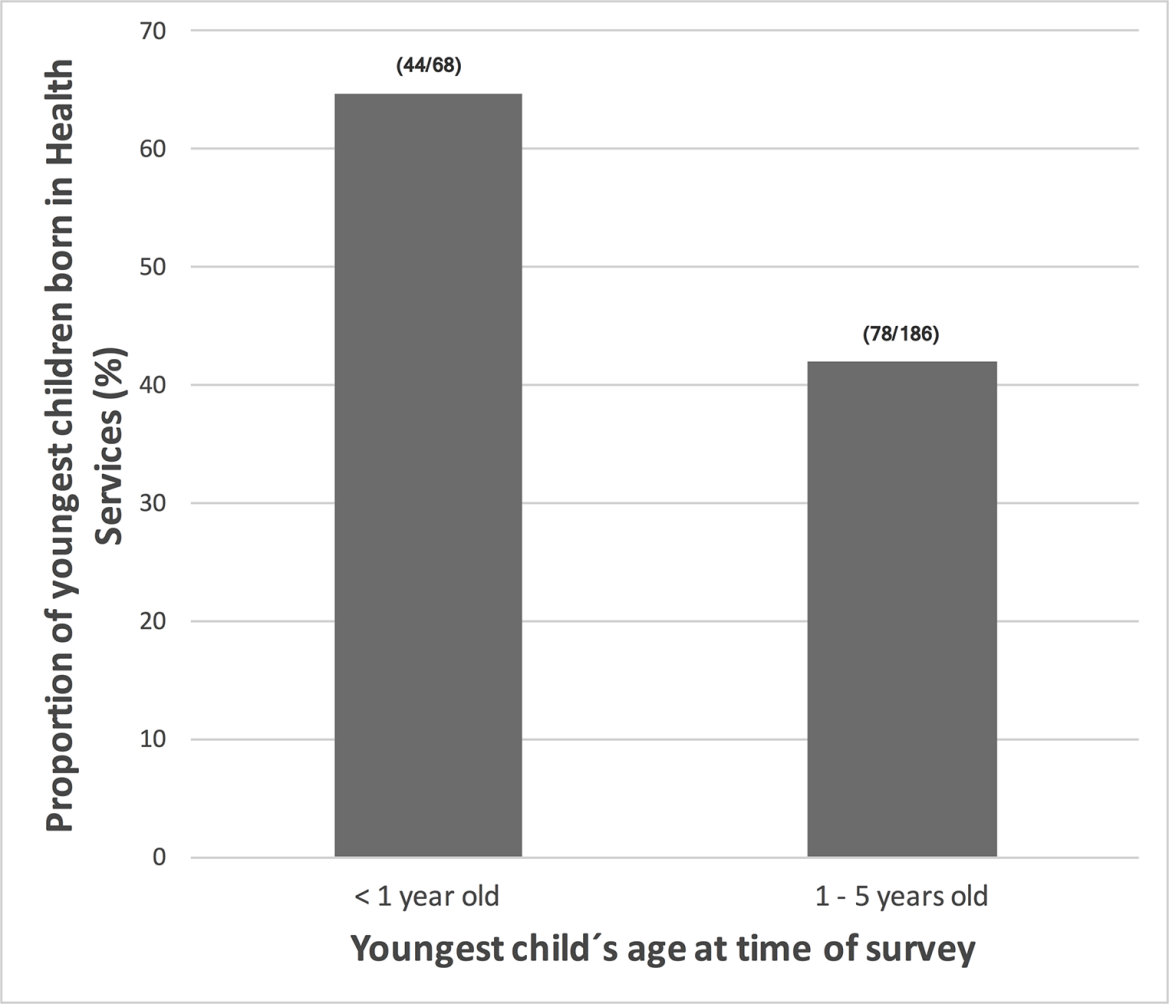
Increased childbirths assisted by health services at Comapa´s Health Center over time. We recorded the number of deliveries within and outside of health services (HS) for children <1 year of age or 1–5 years of age at the time of the health access survey in 2015. Reports were for children born to women living in 18 communities of Comapa with persistent triatomine infestation (n = 490).

The house-to-house survey revealed that almost 50% (240/490) of all women had at least one delivery in a health service facility and only 11% (27/240) were assisted at Comapa´s HC (Table 2). In fact, 81% (401/490) of women reported at least one delivery outside a health facility. Among those whose last delivery was outside a health service facility, 84% (335/401) were assisted by a midwife (Table 2). However, the new maternity ward is a factor enabling the access to maternal health care, with a higher proportion of deliveries of children under one year of age (at the time of the survey) assisted by the health services facilities (Fig 4).

**Table 2:** Attention of deliveries inside or outside health services, health centers or hospitals, according to the health access survey of women from 18 communities with persistent *T*. *dimidiata* infestation in Comapa, Jutiapa, 2015.

Health services questions*	No. positive/Total No. (%)
Had at least one delivery outside the services of a HC or Hospital of Guatemala	401/490 (81.8)
Last delivery at own household	391/401 (97.5)
Last delivery outside the health services, assisted by a midwife	335/401 (83.7)
Last delivery outside the health services, unassisted	39/401 (10.0)
Last delivery outside the health services, assisted by household relative	18/401 (4.0)
Had at least one delivery in a HC or Hospital of Guatemala	240/490 (49.0)
Had at least one delivery at Comapa HC	27/240 (11.0)
Last delivery at Comapa HC	23/240 (9.6)
Had at least one delivery at Jutiapa Regional Hospital	192/240 (80.0)
Last delivery at Jutiapa Hospital	176/240 (73.0)
Had at least one delivery at another HC or Hospital	49/240 (20.0)
Last delivery at another HC or Hospital	41/240 (17.0)

#### Phase 4: Administrative and policy assessment and intervention alignment to identify resource availability for congenital Chagas disease screening

A collaborative multi-stakeholder strategy was developed in 2014 by our research team, IBERMED and the MoH epidemiology and laboratory diagnostics program in Jutiapa to improve the promotion of prenatal screening, congenital disease diagnosis, cardiologic evaluations for adults and treatment of children. After the meetings in March 2014, we performed a new stakeholder analysis in 2016. Through this, we established an alliance with World Vision (NGO with a local program that promotes family well-being by empowering women in Comapa) and the Women´s Municipal Office to promote educational material regarding Chagas disease, transmission routes, prevention and treatment.

In 2015, the interviews with the maternity ward nurse and HC technician revealed that the HC had a prenatal screening program in collaboration with IBERMED since 2014. However, no protocol had yet been established for neonatal screening and no child had been tested for congenital disease yet. Archival evidence reviewed from January 2014 through May 2015 showed that more than half of the births were still assisted by midwives, with midwives assisting 270 of 462 (58%) and the maternity ward assisting 192 of 462 (42%) registered births (Table 3). Laboratory records at the HC showed only eight of 228 (3.9%) pregnant women as Chagas seropositive.

**Table 3:** Deliveries assisted by registered midwives and MoH personnel according to records from the Comapa Health Center, 2014 and 2015.

Assisted delivery system	No. reported deliveries in January-December 2014 (%)	No. reported deliveries in January-May 2015 (%)
Registered midwives*	200 (61.2%)	70 (51.9%)
Comapa Health Center**	127 (38.8%)	65 (48.1%)
Total	327	135

*Registered midwives served 18 communities in 2014 and 11 communities in 2015. Records of midwife-assisted deliveries are missing for February and August of 2014.

**The health center services 38 communities.

#### Phase 5: Intervention design and implementation

**Fig 5:**
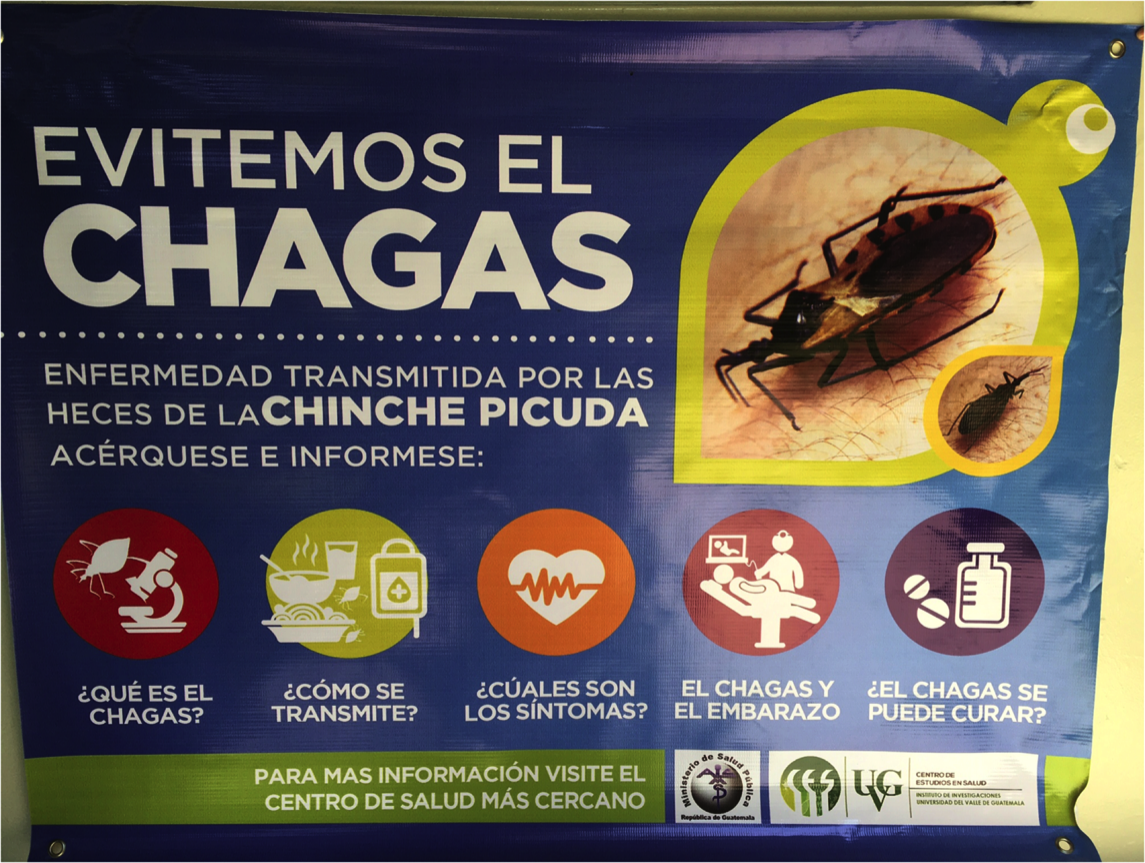
Banner used at Comapa´s Health Center maternity ward to promote congenital Chagas disease screening. An educational flip-chart was developed together with the banner to communicate Chagas disease risk factors, diagnosis and treatment services at the Health Center.

We established a stakeholder-driven strategy that included (1) a promotional program regarding congenital Chagas disease detection for midwives and HC maternity ward personnel and (2) a community-based educational program for women, developed in collaboration with NGOs, the Women´s Municipal Office and the MoH vector control program. In April 2016, a total of 76 flipcharts and banners were distributed to World Vision (26), HC (10), Vector control (12), Women´s Municipal Office (2) and one poster per institutional stakeholder (Fig 5). These promotional and educational materials encourage pregnant women to visit the HC for prenatal Chagas diagnostics. Women with a positive result are informed of the advantages of having the delivery at the HC to provide on-site parasitological diagnostic for the newborn. They are also reminded of newborn screening during the first post-natal visit to the HC. The strategy includes newborn screening on site by heel prick and buffy coat microscopy of a spun microhematocrit to detect mobile *T*. *cruzi*, either after delivery at the HC or during their first tuberculosis/hepatitis B vaccination visit in the first two weeks after birth.

From April to August 2016, seven of eight newborns delivered by seropositive women were screened at the HC, with no positive congenital cases. Two were delivered and immediately tested parasitologically at the HC, five were born outside the HC and tested parasitologically at the HC one week after birth. On August 2016, ongoing educational activities by World Vision and HC personnel were confirmed by collaborators. All parasitology-positive newborns will be offered treatment immediately by the attending nurse, according to the Argentinean National Guideline for pediatric treatment [3]. A registry is kept at the HC, including information regarding diagnosis and treatment. For infants of serology-positive mothers who are not tested at birth, a serology test will be recommended at 10 months of age [28], during their measles mumps rubella vaccination, as well as for children with negative microscopic exams conducted during the perinatal period.

## Discussion

Maternal-infant care in Comapa, Jutiapa, involves public health services, midwives and NGOs. We implemented a multi-stakeholder strategy for neonatal screening to offer timely diagnostics and treatment of congenital Chagas disease. The strategy was generated at the local level through a process including participatory activities with midwives and HC personnel, followed by community-based health communication and educational programs regarding Chagas disease management.

To allow newborn screening and early treatment, the strategy requires (1) reaching the population at highest risk for infection through a community-based health communication program, (2) inclusion of midwives, clinic personnel and NGOs in the implementation of promotional materials for early diagnosis at the HC and (3) HC personnel trained to (a) take newborn blood samples, (b) perform a simple microscopic method to detect parasites in the blood sample, and (c) provide treatment and follow-up for infected neonates. The strategy takes into consideration current maternal-infant care policies and practices at the HC in Comapa, with inclusion of regional NGOs. It also takes advantage of the role played by midwives in informal maternal-infant care, as well as the current national policy requiring their training.

Before our study, infants born to positive women were not screened. To promote newborn detection and treatment, education of midwives and women 15–45 years of age must be developed in a culturally appropriate way. The participatory meetings allowed the development of a socioculturally appropriate strategy for the promotion of congenital Chagas disease screening and treatment in the region. The newborn screening procedure was designed to have a low cost, requiring only microscopic evaluation of the newborn´s blood. The PCR was proposed to confirm microscopic results during method implementation. Once optimized, the parasitological method alone could be implemented in other endemic areas with high seroprevalence. Despite the limitations in maintaining trained personnel in rural areas, we propose that the microscopic method has a potential for sustainability due to its low cost, and could become a standard of care for newborns in these regions. However, rapid test based on the detection of *T*. *cruzi* IgM antigen would be better and should be considered as an alternative once available. On the other hand, the training procedures with midwives can become part of the ongoing program to improve maternal-infant health in the country. Cost estimations have not been included given that all procedures can be implemented without additional expenditure to ongoing activities at the Health Center. Limitations of the proposed strategy will likely include the sustainability of the community-level education programs to promote maternal-infant follow-up visits, the inclusion of the program in current prenatal screening programs such as HIV and syphilis, and the ability to maintain HC competency in parasitological diagnosis and record keeping [29]. As observed in South America [30], social and technical constraints in Chagas disease management in Guatemala include lack of knowledge on the disease, loss to follow up, side effects that lead to treatment non-adherence, lack of communication between decentralized health system levels and lack of training on diagnostics and treatment. We propose that the inclusion of midwives as empowered stakeholders has resulted in referral of newborns to the health center. Future studies will evaluate the strengths and limitations of this strategy, and recommended improvement.

The scaling up of the strategy will require a train-the-trainer program targeting reproductive health and nurse coordinators at the department level for prioritized areas. In addition, evidence of local transmission and education campaigns are needed to empower stakeholders at all levels. Targeted communication campaigns should be developed based on in-depth knowledge of the sociological and cultural behavior of the communities regarding maternal and neonatal care, and how they interact with the health authorities. Forms for recording screening and treatment of mothers and neonates must be developed or modified, and methods of reporting to epidemiological, vector control and policy authorities strengthened. A supply of treatment medication must also be ensured.

The treatment of *T*. *cruzi*-infected women after delivery to reduce the risk of congenital transmission remains a challenge because there are no guidelines regarding treatment during the lactation period. Women with Chagas disease can breast feed, unless they are in the acute phase with high parasitemia, reactivated disease or have bleeding nipples [31]. In rural areas where women have multiple pregnancies, treating infected women during lactation would allow completion of the two-month course before another pregnancy [32]. Research in this area is needed to provide evidence. Finally, treatment before the first pregnancy reduces the risk of congenital transmission [33] and should be considered in future prevention strategies.

A 3.9% seroprevalence in pregnant women attending the HC indicates that early congenital detection and treatment should be a priority in areas with similar historically high triatomine infestations [34] and seroprevalence [14] in Guatemala. To achieve elimination, more studies are needed to understand the prevalence of congenital disease in such populations. In similar areas with persistent triatomine infestation, the MoHs must ensure that Chagas disease control and prevention programs integrate innovative vector control strategies and attention to treatable congenital disease. Future assessment of the strategy is needed to ensure its long term effectiveness and sustainability. The strategy could be expanded to other congenital diseases by strengthening the network of midwives and maternity ward personnel through training in symptom detection at the community level and case referral to health facilities in areas with low access to health services. Given the recent emergence of Zika as a new vector-borne congenital disease, we propose that this stakeholder driven strategy could be implemented in areas with limited access to maternal-infant health services.

## Supporting information

S1 Appendix Database file. (XLSX)
